# Flat Multi-Wavelength Brillouin Erbium-Doped Fiber Laser Based on a Sagnac Loop for High-Sensitivity Sensor

**DOI:** 10.3390/s22229017

**Published:** 2022-11-21

**Authors:** Liang Chen, Jian He, Yi Liu

**Affiliations:** State Key Laboratory of Dynamic Testing Technology, School of Instrument and Electronics, North University of China, Taiyuan 030051, China

**Keywords:** high-sensitivity sensor, flat multi-wavelength fiber laser, Sagnac loop with unpumped erbium-doped fiber

## Abstract

We have demonstrated the use of a flat multi-wavelength Brillouin erbium-doped fiber laser (MWBEFL) based on a Sagnac loop with an unpumped erbium-doped fiber (Un-EDF) as a high-sensitivity sensor. A Sagnac loop with a Un-EDF was used as power equalizer to achieve multi-wavelength power flatness by adjusting the birefringence beat length properly. In the experiments, the best result obtained in terms of Brillouin Stokes lines and output power flatness was ±0.315 dB and the optical signal-to-noise ratio (OSNR) was 18.97 dB within a 33 nm bandwidth range from 1532.0 nm to 1565.0 nm. The flatness of the 33 nm bandwidth range varied from ±0.315 dB to ±1.38 dB and the average OSNR was about 17.51 dB. The peak power values of Brillouin Stokes lines observed under different wavelengths were extremely close and their range of fluctuation was about ±0.37 dB. These experimental results were close to our previous experimental values obtained using a passive Sagnac loop with a Un-EDF. The flat range covering almost the entire C-band has broad application prospects in high-sensitivity distributed optical fiber sensing and wavelength-division multiplexing.

## 1. Introduction

Multi-wavelength fiber lasers (MWFLs) are widely used in wavelength-division multiplexed optical networks, optical device testing, and especially in fiber sensing systems and microwave generation because of their narrow linewidth, low threshold, and stable wavelength interval. In recent decades, a variety of methods have been proposed to obtain MWFLs [1,2,3,4,5,6,7,8,9,10,11,12,13,14,15,16,17,18,19,20], one of which includes the use of stimulated Brillouin scattering (SBS) in the fiber to generate a multi-wavelength Brillouin erbium-doped fiber laser (MWBEFL). Moreover, erbium-doped fiber is often used as a gain medium in erbium-doped optical fiber amplifiers (EDFAs) [21] to provide linear gain for the laser cavity in MWBEFLs [1,2,3,4,5,6,7,8,9,10], and mixing with the nonlinear gain provided by the single-mode fiber (SMF) is carried out to compensate for the laser cavity loss.

In previous studies, MWBEFLs were implemented using different methods, including the use of the Brillouin pump (BP) pre-amplification technique within the linear cavity to obtain an MWBEFL [22], the use of a 70 m highly nonlinear photonic crystal fiber as the Brillouin gain medium to realize a simple MWBEFL [23], using a Sagnac loop filter with polarization-maintaining fiber to achieve a wide-range MWBEFL [5,6], adjusting the polarization controller (PC) in the main laser cavity to modify the characteristics of the polarization-dependent cavity [24], and changing the central wavelength of the BP [25,26,27]. Although these schemes have obtained more wavelengths in the bandwidth range, the output powers of the adjacent Brillouin Stokes lines are quite different in these systems, and the narrow bandwidth has limitations in some practical applications, such as high-sensitivity distributed optical fiber sensing and wavelength-division multiplexing.

In recent years, researchers have focused on achieving power flatness in an MWBEFL. This is due to the lack of a flat design for MWBEFLs, which could have a great impact on certain application fields, such as optical communication systems and optical fiber sensing systems, resulting in a large difference in the output power of Brillouin Stokes lines. In the early research, some researchers used Rayleigh scattering to assist in achieving a flat multi-wavelength output within the 3 dB range and an average optical signal-to-noise ratio (OSNR) of about 17 dB [28]. In further studies, researchers used the combined structure of the ring cavity assisted by the four-wave mixing (FWM) effect and a Brillouin mirror with feedback in 2017, and obtained a flatness of about 4.65 dB within a 16 nm bandwidth in their experiments [29]. In 2018, through the use of strong feedback to enhance the cascaded FWM effect and the self-flattening effect in highly nonlinear fiber (HNLF), and through the introduction of a micro-cavity for flatness optimization, finally a flat output within the 3 dB range within a 14 nm bandwidth was obtained [30]. In a previous study, we found that a Sagnac loop with a Un-EDF can flatten the amplified spontaneous emission (ASE) gain spectrum, and its application to a tunable multi-wavelength Brillouin laser was thus expected to achieve power flatness [31].

In this paper, a flat MWBEFL is proposed based on a Sagnac loop with a Un-EDF, which is regarded as power equalizer, to achieve multi-wavelength Brillouin Stokes power flatness. In our experiments, the output spectrum was flattened by adjusting the birefringence beat length properly in the Sganac loop. Finally, the best flatness value and an OSNR of approximately ±0.315 dB and 18.97 dB were obtained, respectively. Moreover, multiple wavelength experiments in the 33 nm range showed similar flatness effects. The power flatness at different wavelengths was about ±0.315 dB to ±1.38 dB at wavelengths from 1532.0 nm to 1565.0 nm. The average OSNR was about 16.26 dB to 18.97 dB with a 2.7 dB fluctuation. The peak power difference was only ±0.37 dB. Research has shown that the Stokes sensitivity of the K-th order is K times that of the first order [32], and OSNR also affects sensitivity and detection capability. The application of the Sagnac loop with a Un-EDF in multi-wavelength lasers improved the values of Stokes lines, OSNR, and flatness, thereby improving the sensitivity and stability of high-sensitivity distributed optical fiber sensing systems.

## 2. Experimental Setup and Principles

### 2.1. Experimental Setup

**Figure 1:**
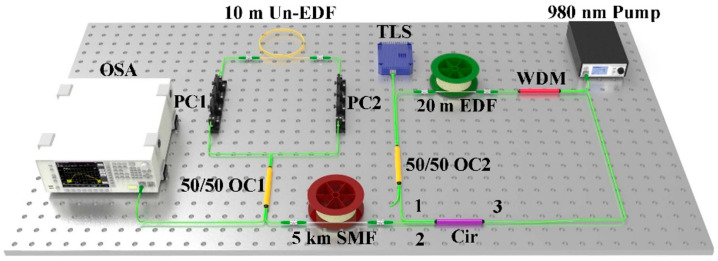
Schematic diagram of the flat MWBEFL based on the use of a Sagnac loop with a Un-EDF. WDM: wavelength-division multiplexer. TLS: tunable laser source. PC: polarization controller. OC: optical coupler. Cir: three-port circulator. OSA: optical spectrum analyzer.

The structure of the proposed flat MWBEFL based on a Sagnac loop with a Un-EDF is shown in Figure 1. The laser includes an EDFA, a tunable laser source (TLS), a three-port circulator (Cir), an SMF, and a Sagnac loop with a Un-EDF. The TLS pump has an adjustable wavelength ranging from 1527.0 nm to 1565.0 nm and an output power ranging from 6 dBm to 14 dBm with a 100 kHz linewidth. A 980 nm pump light source with a maximum power of 500 mW is used to provide energy, which consists of an EDFA with a 980 nm/1550 nm wavelength-division multiplexer (WDM) and a 20 m erbium-doped fiber to provide linear gain to compensate for the optical signal loss. The Brillouin nonlinear gain is provided by a 5 km SMF. The TLS pump is injected into the laser cavity by means of a 50/50 optical coupler (OC2), which is amplified by means of EDFA as a BP light. The Sagnac loop consists of the 50/50 OC1 with a 10 m Un-EDF and two PCs. For the Cir, one of the two ports below the 50/50 OC2 is connected to port 1 of the Cir (the other port is a closed port which is not connected to other devices). The Cir port 2 is connected to the 5 km SMF and Cir port 3 is connected to the 1550 nm port of the WDM. The resolution bandwidth of the spectrum analyzer is set to 0.03 nm, the video bandwidth is set to 1 kHz, and the sampling point is 1001 pt. The TLS pump, injected through the 50/50 OC2, is amplified by means of EDFA in the ring cavity and propagates to the SMF through port 2 of the Cir. When the amplified BP light power exceeds the SBS threshold of the SMF, the first-order Brillouin Stokes (BS1) signal is created. The wavelength separation between the generated Stokes signal and the BP signal light is about 0.08 nm (10.8 GHz). Afterwards, BS1 propagates from port 2 to port 3 of the Cir and is again amplified by means of EDFA, and then continues to propagate in the ring cavity. When the SBS threshold condition is met, BS2 is generated. This process continues until the power of the Stokes lights is insufficient to generate the next-order Stokes lights. When multi-order Stokes light is generated, it is referred to as Brillouin lines.

The input light is transmitted from Cir port 1 to Cir port 2 and flows into the Sagnac loop after the 5 km SMF. Part of the input light is reflected by the Sagnac loop formed by the 50/50 OC2; the other part is output to the optical spectrum analyzer (OSA) for observation through the transmission port. The reflection and transmission can be modified by adjusting PC1 and PC2. The profile thus determines the location of the TLS central wavelength. At the same time, since the Brillouin Stokes passes through SMF twice, the generated bidirectional Brillouin Stokes light significantly enhances the stimulated Brillouin scattering in the SMF. The feedback of light into the cavity from Cir port 3 continues to be amplified by the EDFA to form a cascade to achieve multi-wavelength output.

### 2.2. Principle

As shown in Figure 1, the principle of the use of a Sganac loop with a Un-EDF is as follows: When the incident light is injected from the reflection port of the Sagnac loop, it is divided into two orthogonal beams of opposite transmission. The two beams of light are transmitted to the fast and slow axes of the Un-EDF by adjusting the rotation angle of the PCs respectively, and the difference in the refractive indices of the fast and slow axes is changed to produce a phase difference. The phase difference change causes the birefringence beat length to change in the Sagnac loop, meaning that we obtain different birefringence wavelength intervals, that is, free spectral ranges (FSRs). Finally, the output power of the Brillouin Stokes light is flattened due to the combination of the absorption effect of the Un-EDF and the FSR of the Sagnac loop in the resonance mode of the Sagnac loop. The length of fiber in which the birefringence change causes a periodic change (2π phase difference) is called the birefringence beat length. The birefringence wavelength interval (the FSR) of the Sagnac loop is given by

(1) $$FSR=\frac{\lambda^{2}}{\left(\left|n_{0}-n_{e}\right|\right)L}$$

where $n_{o}$ and $n_{e}$ are the refractive indices of the fast and slow axes of the erbium-doped fiber, $\left|n_{o}-n_{e}\right|$ is the refractive index difference, and L is the length of the Un-EDF in the Sagnac loop.

It can be seen in Equation (1) that when the refractive index difference changes, *FSR* also changes, with an inverse proportional relationship. The Un-EDF is a single-mode fiber doped with erbium and its refractive index difference was about 10^−5^ to 10^−6^ in the experiments. The calculated birefringence and *FSR* ranges were 0.15 m to 1.5 m and 24 nm to 240 nm, respectively. When B gradually decreased, the gain flatness range of ASE became wider. When $B=5\times 10^{-6}$, *FSR* was about 36.5 nm.

**Figure 2:**
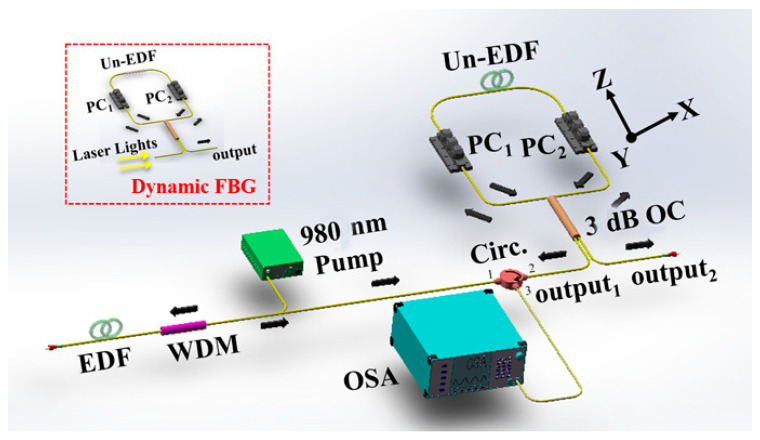
Experimental setup of the gain-flattened EDFA with a Un-EDF.

In previous studies, we demonstrated the use of a Sagnac loop with a Un-EDF for the flattening of the ASE gain spectrum [28]. The experimental setup is shown in Figure 2, and the ASE gain spectrum of the reflection port and the transmission port of the Sagnac loop are studied in detail.

**Figure 3:**
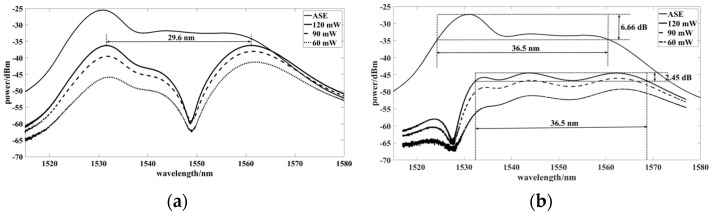
Comparison diagram of ASE gain spectra before flattening and after flattening at the reflection port (**a**) and at the transmission port (**b**).

The gain spectra of the reflection port and transmission port are shown in Figure 3a,b, respectively. The ASE gain spectrum of the transmission port could be flattened to ±1.225 (2.45 dB) by adjusting the PCs properly in the loop, and the wavelength range reached 36.5 nm from 1532 nm to 1568.5 nm. Meanwhile, the ASE gain spectrum of the reflection port could not be flattened by adjusting the PCs properly in the loop, and the ASE spectrum was divided into two peaks with equal power.

## 3. Experimental Results and Discussion

**Figure 4:**
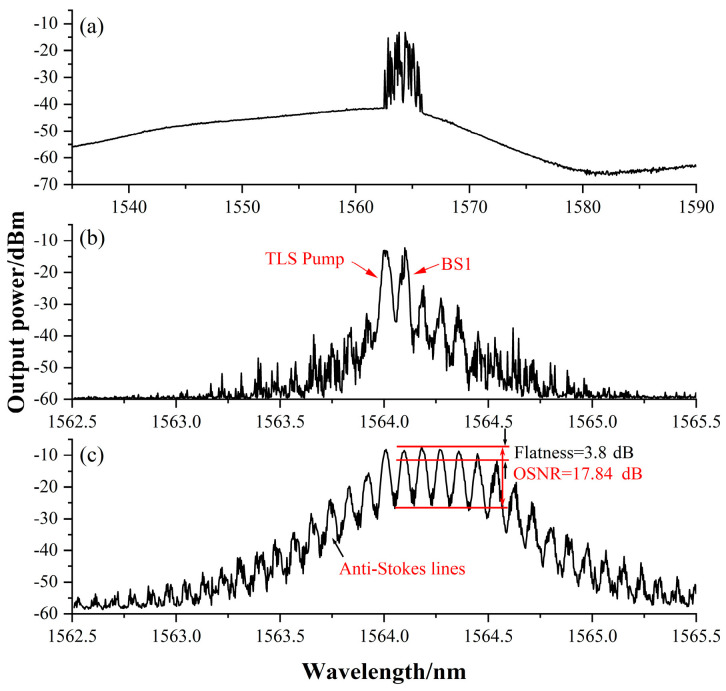
(**a**) ASE and self-excited light without the use of the TLS pump; (**b**) the output spectrum obtained when the 980 nm pump power was 54 mWl (**c**) the output spectrum obtained when the TLS pump power was 14 dBm and the 980 nm pump power was 500 mW.

The ASE generated by means of EDFA without the TLS pump injected into the cavity is shown in Figure 4, and this was operated in the gain range of the EDF (the typical value is 1530–1570 nm). The self-excited output spectral range was about 1562 nm to 1566 nm and the 980 nm pump power was 60 mW. The uniform broadening characteristics of the erbium-doped fiber triggered fierce mode competition and led to unstable transmission, and the range and intensity of the self-excited output were determined by the ion concentration of the EDF, the 980 nm pump power, and the cavity loss. By setting the TLS pump’s central wavelength within the self-excited range, we were able to effectively suppress mode competition to obtain a better multi-wavelength output. On the contrary, when the TLS pump wavelength was far from the self-excited range, the linear gain of EDFA was absorbed because self-excitation in the cavity could not be effectively suppressed. Low-order Stokes light could not be fully amplified and high-order Stokes light could not be generated because the Brillouin threshold had not been exceeded. Moreover, mode competition was intensified when the gain of the EDFA increased and higher TLS pump power was required to suppress the self-excited output. Figure 4a depicts the output spectrum at a certain moment. As shown in Figure 4b, the TLS pump power was set to 6 dBm and the 980 nm pump power was increased from 0 mW to 54 mW. The TLS pump power exceeded the SBS threshold and a first-order Brillouin Stokes signals (BS1) began to appear at an interval of 0.089 nm from the TLS pump and continued to propagate in the opposite direction to the TLS pump. If the TLS pump power and EDFA power were increased, the TLS pump and BS1 would continue to circulate in the cavity and then be amplified by the EDFA to generate BS2 and higher-order Stokes signals until their power became insufficient.

As shown in Figure 4c, when the TLS pump power was increased to 14 dBm and the 980 nm pump power was increased to 500 mW, the number of Stokes lines increased to 6, and the flatness was 3.8 dB. Under this experimental scheme, the main factor affecting the number of Stokes lines was the 980 nm pump power (maximum 500 mW). If an EDFA with higher gain was used, more Stokes lines could be saturated, and the absorption of optical power by the Un-EDF and the peak power of the Stokes lines would become flatter.

**Figure 5:**
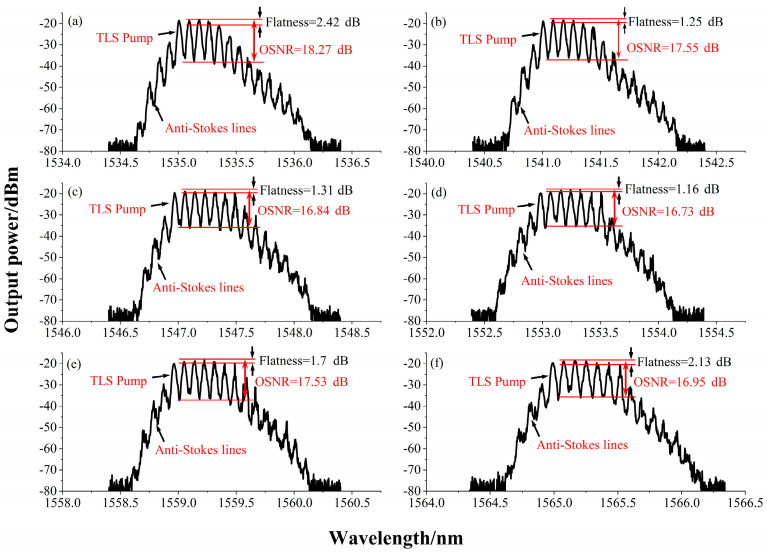
Output spectra of different TLS wavelengths. (**a**) TLS wavelength = 1532 nm, (**b**) TLS wavelength = 1542.5 nm, (**c**) TLS wavelength = 1547 nm, (**d**) TLS wavelength = 1553 nm, (**e**) TLS wavelength = 1559 nm, (**f**) TLS wavelength = 1565 nm.

To study the flatness of the output power in the entire flat range, the TLS wavelength was set to six different conditions ranging from 1532 nm to 1565 nm. After setting the TLS wavelength for the first time and flattening the output power by adjusting the two PCs, the states of the two PCs remain unchanged after changing the TLS wavelength again. As shown in Figure 5, the TLS pump wavelength was set at 1532 nm, 1542.5 nm, 1547 nm, 1553 nm, 1559 nm, and 1565 nm, and the peak power of the output spectrum was −18.19 dBm, −18.25 dBm, −18.49 dBm, −18.18 dBm, −18.30 dBm, and −18.75 dBm, respectively. The peak power difference obtained at different central wavelengths was only 0.57 dB; the power flatness ranged from 0.63 dB to 2.76 dB. The number of relatively flat spectral output lines obtained at different wavelengths ranged from approximately four to six, and the power level of the Brillouin Stokes lines between adjacent channels was extremely close to the wavelength interval. The best output power flatness achieved throughout the experiment was 0.63 dB (±0.315 dB), obtained when the TLS wavelength was 1532 nm. The worst power flatness obtained throughout the experiment was 2.76 dB (±1.38 dB), which was observed when the TLS wavelength was 1542.5 nm. Furthermore, we could clearly see the existence of anti-Stokes lines, and the right side of the higher-order Stokes signal included lines that could not exist stably due to unsaturated gain.

**Figure 6:**
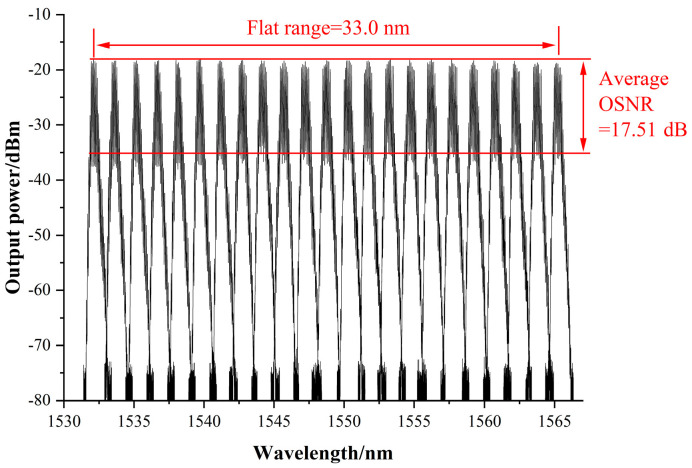
Flat output spectra of different TLS wavelengths in the 33 nm range.

Next, the TLS pump’s central wavelength was set from 1532 nm to 1565 nm with 1.5 nm as the step unit to explore further the flatness effect of the use of a Sagnac loop with a Un-EDF in the entire bandwidth. The 980 nm pump power was 500 mW, and the TLS pump power was constant at 14 dBm. The Brillouin Stokes output spectrum was flattened by adjusting PC1 and PC2 when setting the TLS wavelength for the first time, maintaining the two PC states and changing the TLS wavelength and recording 23 experiments with OSA. As shown in Figure 6, there was almost no difference in the Brillouin Stokes lines’ output power at different TLS wavelengths. The average OSNR of all Stokes lines was 17.51 dB.

**Figure 7:**
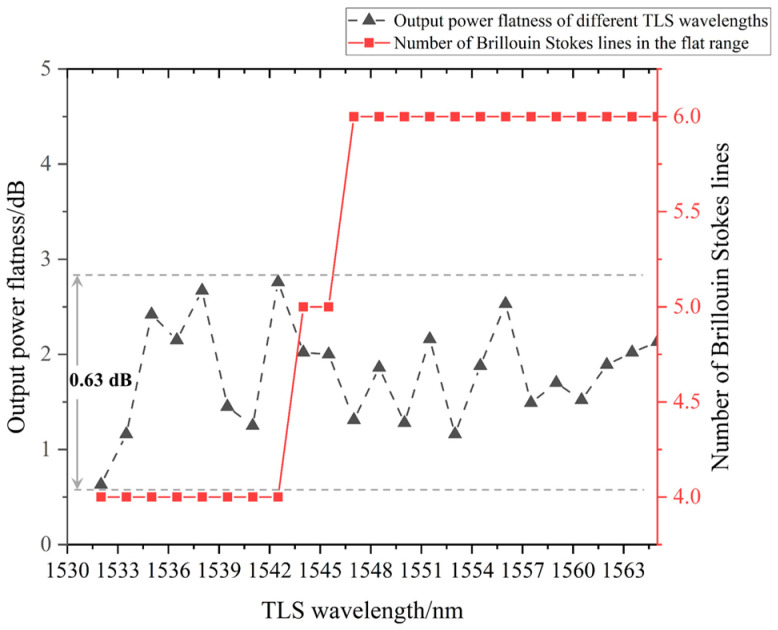
Output power flatness at different TLS wavelengths and the number of Brillouin Stokes lines in the entire flat range.

**Figure 8:**
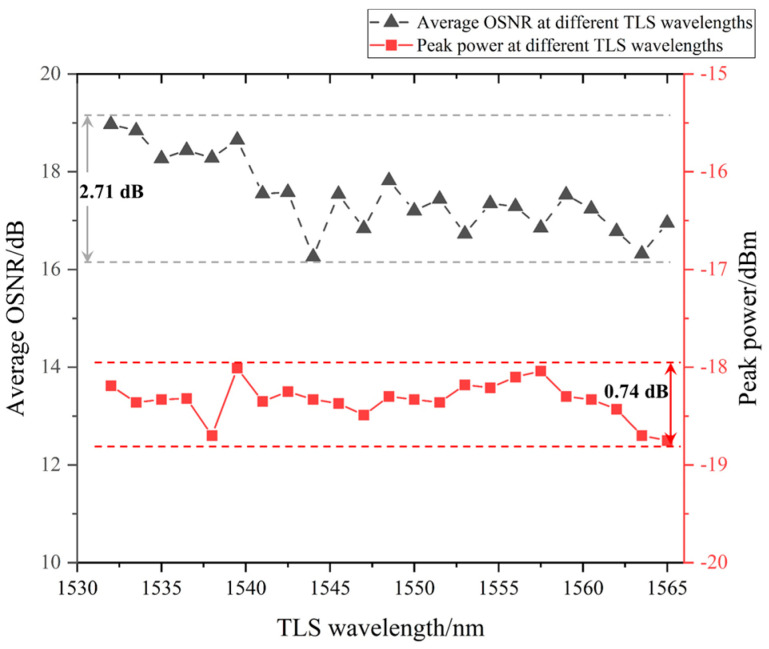
Brillouin Stokes lines’ average OSNR and peak power at different TLS wavelengths.

The results of 23 experiments were statistically analyzed to obtain the power and number of multi-order Stokes lines with stable power output in each experiment. The peak power of each order of Stokes lines was recorded to obtain its flatness and OSNR. According to the peak power of the Stokes lines in each experiment, the peak power fluctuations observed in multiple experiments were counted, and these are illustrated in Figure 7 and Figure 8. Figure 7 shows the number of effective Brillouin Stokes lines in the flat range and the power flatness when the TLS pump’s central wavelength was set at different wavelengths. The numbers of Brillouin Stokes lines, as shown in Figure 7, increased as the TLS wavelength position increased in steps from 1532.0 nm to 1565.0 nm. The output power flatness values obtained under different TLS wavelengths were relatively close, and there were no large fluctuations. The output power flatness ranged from approximately ±0.315 dB to ±1.38 dB (0.63 dB to 2.76 dB). These values were very close to our previous passive experimental values, and the overall flattening effect of the Sagnac loop with the Un-EDF for ASE gain was about 2.45 dB.

As shown in Figure 8, there was only a 0.74 dB (±0.37 dB) difference in the peak power in 23 experiments at different TLS wavelengths. The highest peak power was −18.01 dBm and the lowest peak power was −18.75 dBm. In most cases, the peak power showed a difference of only 0.2 dB (−18.30 dBm to −18.50 dBm). The average OSNR at different TLS wavelengths ranged from 16.26 dB to 18.97 dB, with a 2.71 dB fluctuation. These analyses further proved that the Sagnac loop with a Un-EDF had a flattening effect on multi-wavelength output power. The flattening effect was basically able to cover the entire flat range, which was approximately equal to the C-band.

## 4. Conclusions

In conclusion, we investigated the power flatness of a flat MWBEFL based on the use of a Sagnac loop with a Un-EDF. In our experiments, we finally obtained multi-wavelength Brillouin Stokes lines with a power flatness performance of ±0.315 dB to ±1.38 dB in 23 different central wavelength experiments, with a flat range of 33 nm. The OSNR ranged from 16.26 dB to 18.97 dB and the overall average OSNR was calculated to be about 17.51 dB. The peak power fluctuation range was −18.01 dBm to −18.75 dBm. Although the maximum number of Brillouin Stokes lines obtained using our design was only six, we achieved a power flatness with a 33 nm range, covering almost the entire C-band. If we adopt other MWBEFL structures with the aim of realizing power flattening using a Sagnac loop with a Un-EDF, the laser’s flatness is expected to be improved. The flatness and flexibility of a flat MWBEFL can be applied to high-sensitivity distributed optical fiber sensing and wavelength-division multiplexing.

## Data Availability

Not applicable.
